# Carbon Pool in Mexican Wetland Soils: Importance of the Environmental Service

**DOI:** 10.3390/life12071032

**Published:** 2022-07-11

**Authors:** Sergio Zamora, Irma Zitácuaro-Contreras, Erick Arturo Betanzo-Torres, Luis Carlos Sandoval Herazo, Mayerlin Sandoval-Herazo, Monserrat Vidal-Álvarez, José Luis Marín-Muñiz

**Affiliations:** 1 Facultad de Ingeniería, Construcción y Habitad, Universidad Veracruzana, Bv. Adolfo Ruíz Cortines 455, Costa Verde, Boca del Rio 94294, Veracruz, Mexico; szamora@uv.mx; 2 Academy of Sustainable Regional Development, El Colegio de Veracruz, Xalapa 91000, Veracruz, Mexico; izitacuaro@yahoo.com; 3 Wetlands and Environmental Sustainability Laboratory, Division of Graduate Studies and Research, Tecnológico Nacional de México/Instituto Tecnológico de Misantla, Veracruz, Km 1.8 Carretera a Loma del Cojolite, Misantla 93821, Veracruz, Mexico; eabetanzot@itsm.edu.mx (E.A.B.-T.); lcsandovalh@gmail.com (L.C.S.H.); 4 Department of Business Management Engineering, Tecnológico Nacional de México/Instituto Tecnológico de Misantla, Veracruz, Km 1.8 Carretera a Loma del Cojolite, Misantla 93821, Veracruz, Mexico; mayerli.sandoval24@gmail.com

**Keywords:** wetlands, environmental services, carbon soil sequestration, carbon budgets

## Abstract

Mexican wetlands are not included in Earth system models around the world, despite being an important carbon store in the wetland soils in the tropics. In this review, five different types of wetlands were observed (marshes, swamps, flooded grasslands, flooded palms and mangroves) in which their carbon pool/carbon sequestrations in Mexican zones were studied. In addition, it was shown that swamps (forested freshwater wetlands) sequestered more carbon in the soil (86.17 ± 35.9 Kg C m^−2^) than other types of wetlands (*p* = 0.011); however, these ecosystems are not taken into consideration by the Mexican laws on protection compared with mangroves (34.1 ± 5.2 Kg C m^−2^). The carbon pool detected for mangrove was statistically similar (*p* > 0.05) to data of carbon observed in marshes (34.1 ± 5.2 Kg C m^−2^) and flooded grassland (28.57 ± 1.04 Kg C m^−2^) ecosystems. The value of carbon in flooded palms (8.0 ± 4.2 Kg C m^−2^) was lower compared to the other wetland types, but no significant differences were found compared with flooded grasslands (*p* = 0.99). Thus, the carbon deposits detected in the different wetland types should be taken into account by policy makers and agents of change when making laws for environmental protection, as systematic data on carbon dynamics in tropical wetlands is needed in order to allow their incorporation into global carbon budgets.

## 1. Introduction

Atmospheric concentrations of greenhouse gases (GHGs) are at levels unprecedented in at least 800,000 years. Concentrations of carbon dioxide (CO_2_), methane (CH_4_) and nitrous oxide (N_2_O) have all shown large increases since 1750 (40%, 150% and 20%, respectively) [1]. Thus, wetlands are an option to help to mitigate the impact of climate change as they can regulate, capture and store GHGs [2,3] due to their dense vegetation, microbial activity and soil conditions. 

Wetlands are transitional zones between terrestrial and aquatic ecosystems, areas of water saturated with soil that covers about 5 to 8% of the land surface of Earth [2]. They include floodplains with forest or herbaceous vegetation. Wetlands play an important role in the global carbon cycle; they have the best capacity of any ecosystem to retain carbon in the soil, as these ecosystems have a high capacity to limit the availability of oxygen to soil microbes and the decomposition of organic matter [4,5]. 

Decomposition of organic matter within wetlands involves aerobic and anaerobic processes. Organic matter decomposition (shrub residues, detritus, etc.) is often incomplete under anaerobic conditions due to the lack of oxygen [5]. The carbon accumulation formed over time is vulnerable when the wetland soil is affected by five factors, which includes pollution, biological resource use, natural system modification, agriculture and aquaculture [6]. These changes may have important repercussions on global warming. However, despite the fact that approximately 30% of the world’s wetlands are found in the tropics [2,7], only a few studies [7,8] regarding carbon sequestration in wetlands have been conducted and taken into account for the global carbon budget in the tropical regional zone of Mexico. 

Of the total storage of carbon in the earth’s soils (1400–2300PgC (Pg = 10^15^ g of carbon)), 20–30% is stored in wetlands [2]. Some studies around the world on carbon sequestration have shown the importance of natural soil wetlands; for example, North American wetlands contain about 220 PgC, most of which is in peat [8]. Wetlands in the conterminous United States store a total of 11.52 PgC [9]. Another study suggests that the world’s wetlands serve as a net sink of 0.83 PgC/year, estimated from 21 wetland simulations that included Russia, Canada, Costa Rica, Botswana and the USA data on carbon sequestration wetland soils [10]. However, information of Mexican carbon pools was not considered in American data. The knowledge regarding carbon sequestration and storage in all the wetland soils is critical for successful pathways to global decarbonization and carbon budgets.

In 2022, the Convention on Wetlands of International Importance, known as theRAMSAR Convention or RAMSAR treaty, celebrates 51 years of efforts to conserve these crucial ecosystems. The treaty includes 2435 designated wetlands of international importance (Ramsar sites), which involves 254,685,425 hectares of wetland soils around the world (172 contracting parties). Mexico is in second place with wetland Ramsar sites (142) after the United Kingdom (175), covering 8.7 million ha. Many of the priority wetland sites in Mexico are associated with coastal sites on the Gulf of Mexico and the Pacific Ocean [11].

According to this, it is essential to know the Mexican wetlands. Thus, the main objective of this study is to describe the natural wetlands of Mexico, their uses, and soil carbon pool or carbon sequestration function. 

## 2. Materials and Methods

The authors undertook a comprehensive search of the literature on carbon sequestered in Mexican wetland soils (mangroves, flooded palms, swamps, flooded grassland, marshes) based on the most important databases located in Mexican universities, publications of the Mexican carbon program (http://pmcarbono.org/pmc/publicaciones/sintesisn.php (accessed on 5 June 2022)), and the ISI Web of Knowledge (www.isiknowledge.com (accessed on 8 April 2022)) database. The keywords used were: (carbon-pool, -stock, -sinks, -sequestration, soil, wetlands, mangroves, flooded palms, swamps, flooded grassland, marshes using the booleans operators and/or (exclusively in Spanish and English). A total of 482 studies (from the year 2000 to 2022) were identified regarding carbon sinks for Mexican wetland soils; only 32 studies were selected based on studies in situ on carbon pools or carbon sequestration in Mexican wetlands because most were theoretical topics or reviews, of which 56 sites were analyzed because in some papers different types of wetlands were studied. The remaining percentage of studies was used for the introduction section, the justification of the study and the discussion of the data. 

To provide context, it is important to mention that, in most cases, the method formeasuring the carbon sequestered in wetland soils consists of sampling points of soil profiles used to analyze carbon and bulk density. Organic carbon is obtained by analyzing the percentage of organic matter (OM) content and calculated as a portion of OM by using the conversion coefficient of 0.58 (Van Bemmelen factor) [3,7,10] or the factor of 0.50 proposed by Mitsch and Gosselink [2].

Statistical analyses to determine differences in carbon pools among wetland soils were performed with IBM SPSS Statistic version 22 for Windows (Armonk, NY, USA: IBM Corp.). A Kolmogorov–Smirnov test was used to check normality; data fitted no normal distributions; thus, the Kruskal–Wallis test at the 5% significant level was used to find differences in the carbon pools between different wetland types.

## 3. Results and Discussion

### 3.1. Importance of Natural Wetlands to Ecosystem Services

Natural wetlands are important areas in terms of natural resources and biodiversity. The publication of the Millennium Ecosystem Assessment [12] described a categorization for wetland ecosystem services with four types: (1) Provisioning ecosystem services include products obtained from ecosystems, such as food, water, timber, fiber, or genetic resources. (2) Regulating ecosystem services include air quality and climate regulation, water purification, disease/pest regulation, pollination, and natural hazard regulation. (3) Cultural ecosystem services include benefits that people obtain from ecosystems related to spiritual enrichment, recreation, ecotourism, aesthetics, formal and informal education, inspiration, and cultural heritage, and (4) supporting ecosystem services include basic ecosystem processes of nutrient cycling and primary productivity that may, in turn, lead to the other three services listed above.

Considering the ecosystem services described above, wetlands are known as “the kidneys of the landscape” and “ecological supermarkets”, and they provide a potential sink of carbon [2]. Despite the fact that a large percentage of wetlands occur in tropical latitudes, carbon sequestration from coastal tropical Mexican wetlands has not been extensively reported. 

### 3.2. Wetland Types

Natural wetlands include marine, coastal or continental wetland types. The water that flows into the wetland could be freshwater, brackish or saline. According to the vegetation, the wetlands can have herbaceous vegetation or forest plants. However, some specific classifications are described:

Forested wetlands: these are wetlands dominated by trees in temporal or permanent flooded conditions. Wetlands dominated by halophytic trees growing in brackish to saline tidal waters are called Mangroves. Typical mangrove trees are *Avicennia germinans*, *Laguncularia racemosa* and *Rhizophora mangle* L. Wetland trees growing in freshwater conditions are called freshwater swamps. In this study, only the concept “swamp” was used. Typical freshwater swamp trees are *Pachira aquatica*, *Haematoxylum campechianum*, *Ficus insipida*, *Taxodium distichum* and *Nyssa aquatic* [2,13].

Herbaceous wetlands: these are wetlands dominated by emergent herbaceous vegetation adapted to saturated soil conditions. In wetlands with freshwater conditions, common species are *Typha* spp., *Thalia geniculate* L., *Scirpus* spp. and *Pontederia sagittata*. While common species of herbaceous wetlands growing in brackish or saline water are: *Spartina alterniflora, Salicornia quinqueflora* and *Galaxias maculates* [3,13]. In this study, these types of wetlands are considered as marshes (wetlands dominated by herbaceous plants, such as grasses, reeds and sedges [2,3]).

On the other hand, other types of herbaceous wetlands are the flooded grasslands: these wetlands are characterized by an abundance of grass (or sedges), as well as periodic flooding with fresh or brackish water or a high-water level during some months of the year, sufficient to influence the vegetation. Typical grasses include species of the *Poaceae* family or sedges with species of the *Cyperaceae* family [14].

### 3.3. The Carbon Cycle and Dominant Organisms in Wetland Soils 

**Figure 1:**
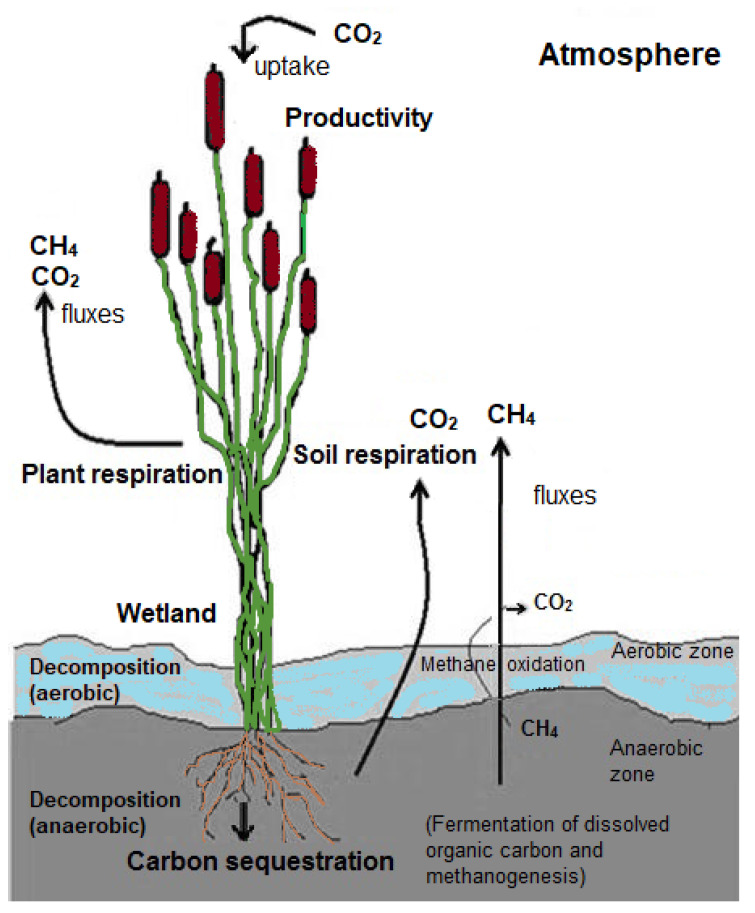
Schematic diagram showing the major components of the carbon budget in a wetland and its carbon exchanges with the atmosphere (adapted from [2,7,10]).

Carbon capture and sequestration is a physical process that involves the capture of atmospheric carbon dioxide (CO_2_) and its storage. In wetlands, the major components of the carbon cycle are illustrated in Figure 1. Various reactions utilizing carbon take place within wetlands. The key processes are respiration and photosynthesis in aerobic conditions, fermentation, methanogenesis, methane oxidation and sulfate, iron, and nitrate reduction in the anaerobic areas [15]. 

Photosynthesis (6CO_2_ + 12H_2_O + light → C_6_H_12_O_6_ + 6O_2_ + 6H_2_O) and aerobic respiration (C_6_H_12_O_6_ + 6O_2_→ 6CO_2_ + 6H_2_O + 12e^−^ + energy) dominate the aerobic areas (aerial and aerobic water and soil), with H_2_O as the major electron donor in photosynthesis and oxygen as the terminal electron acceptor in respiration [2].

The fermentation of organic matter or glycolysis for the substrate involved occurs when organic matter is the terminal electron acceptor in the anaerobic respiration of microorganisms. This forms various low-molecular-weight acids and alcohols, as well as carbon dioxide, e.g., lactic acid (C_6_H_12_O_6_→ 2CH_3_CH_2_OCOOH) and ethanol (C_6_H_12_O_6_→ 2CH_3_CH_2_OH + 2CO_2_). Fermentation represents one of the major ways in which high-molecular-weight carbohydrates are broken down to low-molecular-weight organic compounds, usually as dissolved organic carbon, which is, in turn, available to other microbes [2,16].

The methanogenesis occurs when certain methanogenic bacteria members of the Archaea domain use CO_2_ as an electron acceptor for the production of gaseous methane (CO_2_ + 8H+ → CH_4_ + 2H_2_O). Depending on the wetlands and type of archaea, hydrogenotrophic methanogenesis or acetoclastic processes occur. In non-fertilized soils, it has been observed that acetoclastic methanogenesis represents 51–67% of the produced methane [16]. On the other hand, methane oxidation is carried out by obligate methanotrophic bacteria, which are from a larger group of eubacteria; they convert methane gas in sequence to methanol (CH_3_OH), formaldehyde (HCHO), and finally CO_2_ (CH_4_→ CH_3_OH → HCHO → HCOOH → CO_2_)

Sulfate reduction: this metabolism is carried out by sulfate-reducing bacteria (SRB) when the redox potential (Eh) decreases to −120 mV. SRB use mainly sulfate as their terminal electron acceptor in the anaerobic oxidation of organic substrates and reduce it to hydrogen sulfide (H_2_S) (2(CH_2_O) + SO_4_^−2^ 2HCO^3−^ + H_2_S). Sulfate reducers are capable of using formate, lactate and H_2_; therefore, they compete with methanogens for substrates [2,16]. In coastal sediments, distinct depth distributions have been observed, where SO_4_^2−^ reducers are abundant in the first few centimeters, but as SO_4_^2−^ is depleted, methanogens become more abundant at greater depths [17].

Iron reduction: this is a process carried out when the Eh descends to –47 mV. Several groups of facultative and anaerobic bacteria participate in it. Manganese-reduction and iron-reduction are relevant processes in those wetlands with high mineral supplies [16].

Denitrification: it is a respiration process in which the electron acceptor is nitrate, and it starts when oxygen concentration is <10 µM. The resulting denitrification products are molecular nitrogen (N_2_) and nitrogen oxide (NOx). Anaerobic Gram-negative bacteria performs this process; among them, the genera *Pseudomonas* spp., *Clostridium* spp., *Bacillus* spp. and *Alcaligenes* spp. have been reported [18].

Hydrology and radial O_2_ leakage have differences in the oxidation state of metals. Therefore, site mineralogy interacts with hydrology to shape the wetland microbial community. For example, wetland roots are often coated with iron (Fe) (III) and Manganese (Mn) (IV) oxides. Plants supply electron donors in the form of root exudates and oxidize metals through O_2_ leakage, supporting metal-reducing bacteria [19].

Soils of organic matter typically contain between 45% and 50% carbon. Organic soils formed from plant debris decompose slowly in very wet settings due to low oxygen conditions, also referred to as anaerobic. Organic soils are very black, porous, and light in weight and are often referred to as “peat” or “musk” [20]. Another process in wetlands is respiration, described as the biological conversion of carbohydrates to carbon dioxide, and fermentation is the conversion of carbohydrates to chemical compounds such as lactic acid, or ethanol and carbon dioxide. In a wetland, organic carbon is converted into compounds including carbon dioxide and methane and/or stored in plants, dead plant matter, microorganisms, or peat [15,16]. 

Microbial degradation of above-ground plant litter is likely to begin before the material enters the soil, and the role of fungi in wetlands needs more investigation. Some dominant organisms in wetland soils described in a mini review [19] include fungi (*Chaetothyriales, Cantharellales*), bacteria (*Bacteroides*, *Planctomycetes*, *Chloroflexi*, *Acidobacteria*, *Actinobacteria*), fermenters (*Chloroflexi*, *Proteobacteria*, *Verrucomicrobia*), iron reducers (*Geobacter* sp., *Desulfovibrio* sp., *Anaeromyxobacter*, *Shewanella*), sulfate reducers (*Desulfarculales*, *Desulfovibrionales*, *Syntrophobacterales*, *Firmicutes*, *Desulfobulbaceae*), methanogens (*Methanoregulaceae*, *Methanosarcinaceae*, *Methanosaetaceae*) and methanotrophs (*Methylobacter*, *Methylocystis*, *Methylobacter*).

### 3.4. Carbon Sequestration in Mexican Wetland Soils

Considering that the importance of wetlands in carbon sequestration as an environmental service to mitigate global warming was described by the Millennium Ecosystem Assessment [12], it is important to highlight that this regulating ecosystem service is scarcely perceived or recognized by the population. 

Generally, provisioning and the cultural ecosystem services are the most identified because they are more visible or palpable [21], so the dissemination of this type of knowledge is important, and this study highlights such function, in which it is important to note that even though there are some global carbon balances, counts or earth system models, these do not include Mexican or tropical data [2,9,12,22]. However, in the last 20 years, measurements of the carbon storage function have been made in Mexico, including mangroves, swamps, marshes, flooded palms and flooded grasslands (Table 1). 

Carbon sequestration in Mexican marshes oscillated between 16 and 110 Kg C m^−2^ (Table 1), while data of carbon pool reported for marshes of Old Woman Creek from Ohio were between 9 and 14 Kg C m^−2^. In Palo Verde, a national park in Costa Rica, only 6 to 7 Kg C m^−2^ were reported [23]. The carbon pool observed in the riverine marsh of Botswana, Africa, oscillated within 0.8–1.3 Kg C m^−2^ [24], underlining the importance of Mexican tropical wetlands in carbon storage, in addition to highlighting the importance of these data for the generation of regulations or public policies for their protection since these types of wetlands do not have extensive legal protection as in the case of the mangroves.

**Figure 2:**
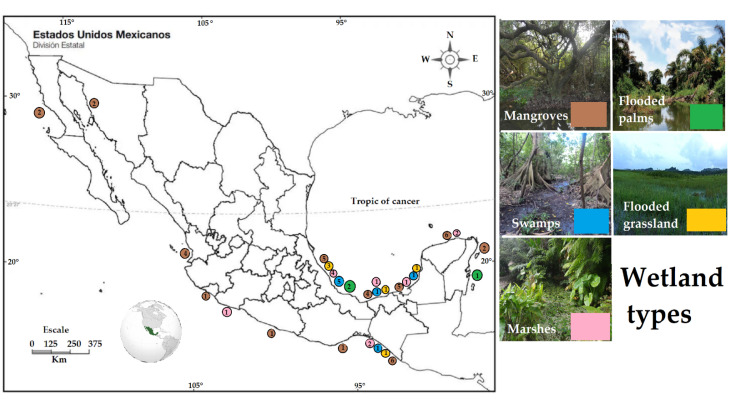
Location of the Mexican wetlands reviewed. Places represented by letters are referenced in Table 1. The number inside the circle is the number of studies in that state/site. The color of the circle represents the wetland type.

The flooded grasslands are sites with minimal attention regarding the function of the carbon pool. In Mexican regions, three studies were found between the states of Veracruz, Chiapas and Tabasco (Table 1, Figure 2), with carbon sequestered in the soil within 28 and 31 Kg C m^−2^. In the same regions, one of the studies mentioned above [25] claims that soil carbon concentration decreases in areas converted from swamps of forested wetlands to flooded grasslands due to decreases in carbon inputs, physical disturbances, and shorter hydroperiods, which enhance higher greenhouse emissions, so the changing land use negatively affects the ecosystem services as a carbon pool.

Regarding mangroves, in Mexico, they are the wetland type with the best policies and law enforcement for protection and conservation [3]. However, despite this importance, mangroves are being deforested. Throughout the twentieth century, 30–50% of global mangrove cover has been destroyed. Using previously published global models of carbon stocks and Mexico-specific carbon sequestration data and calculating gross deforestation, it was found that the current rate of deforestation will result in a social cost of USD 392.0 (±7.4) million over the next 25 years [26]. 

Thus, it is essential to follow up on the policies established in the country and avoid permits for land-use change in these ecosystems, considering that the values of carbon sequestered in mangrove soils oscillate between 7 and 93 Kg C m^2^ in almost all the coastal zone of the country (Table 1, Figure 2). The values reported are similar to data in inventories of soil carbon for natural and replanted mangrove forests from tropical and subtropical areas, including Indonesia, Thailand, China and Australia (28–56 Kg C m^−2^) [27].

Regarding swamps, these ecosystems have been recognized as treasures of the country, and some books have described the importance, history, science and policies of these wetland sites [2,3,28]. In Mexico, for example, some studies have reported their importance as a carbon pool, mainly in the coastal areas of Veracruz and Chiapas with values between 35 and 73 Kg C m^−2^, while in other tropical zones such as Costa Rica or temperate areas such as Ohio, the values reported for similar wetland types are lower (10–21 Kg C m^−2^) [10,23].

Flooded palms are a type of wetland that is less common; however, in Mexico, three sites with carbon pool data were identified in Veracruz and Quintana Roo (Table 1, Figure 2), with values of 1.5 to 16 Kg C m^−2^. These ecosystems are important as the fruits of the palms are widely used for the preparation of traditional recipes, and the stem of the palms is used for house construction [29,30]. At EARTH University, Humedal La Reserva, of Costa Rica, in the middle of the rainforest reserve on the university campus, there is a swamp palm dominated by *Raphia taedigera* with a carbon pool of 15.28 Kg C m^−2^ reported in the soil, similar to the maximum value detected for Mexican flooded palms.

### 3.5. Mean Carbon Sequestration or Carbon Pool in Mexican Wetland Soils

**Figure 3:**
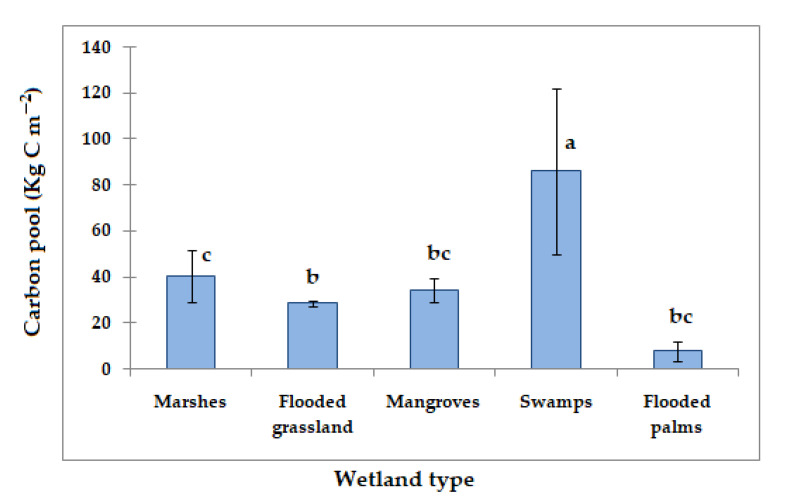
Carbon pool in the different wetland types of Mexico. Lines over the bars are the standard error. Letter over the bars represents statistical analysis (different letters imply values significantly different (*p* < 0.05) form each other).

The use of natural ecosystems to accumulate carbon in the soil is one of the most cost-effective tools for reducing the net effect of greenhouse gas emissions and abating climate change [62]. Mexican wetlands have been studied mainly in the last 10 years regarding their high productivity in organic matter in the soil; the values reported were averaged according to the wetland type (Figure 3), finding statistical differences of (*p* = 0.011). The best wetland type for carbon sequestration in the soil was the swamp with 86.17 ± 35.9 Kg C m^−2^; this value was significantly higher than flooded grassland (28.57 ± 1.04 Kg C m^−2^; *p* = 0.017), mangroves (34.1 ± 5.2 Kg C m^−2^; *p* = 0.010), flooded palms (8.0 ± 4.2 Kg C m^−2^; *p* = 0.017) or the marshes (40.55 ± 11.5 Kg C m^−2^; *p* = 0.049). These values are very important for climate models of the carbon balance. Marín-Muñiz et al. [63] argued that wetlands should be considered as a sink of carbon in the 100-year time horizon. Thus, the importance of conserving and protecting these ecosystems is worth mentioning. 

On the other hand, the carbon pool in mangrove soils revealed significant differences (*p* < 0.05) with respect to the carbon pool of flooded palm and swamp zones. Flooded palm soils sequestered a similar amount of carbon to flooded grassland (*p* = 0.100) but were different compared to the other wetland types. The carbon in marshes was statistically similar to the carbon pool in flooded palms (0.990) and mangrove soils (*p* = 0.447). The importance of the vegetation regarding the quantity of carbon sequestration in the soil has been documented in some studies [31,64]; similarly, other factors such as water level and flooded conditions are also important in the carbon pool in the wetlands [8,31].

Comparing the carbon pool of Mexican wetland soils with other reported values reported of wetlands in other countries or for the Mexican terrestrial ecosystems, it was observed that swamps, marshes, mangroves, and flooded grasslands can store almost 13, 7, 6, and 5 times more carbon in the soil than Mexican terrestrial ecosystems (Table 2), respectively. Only the carbon stored in flooded palm wetlands was similar to the carbon of Mexican terrestrial ecosystems. A similar situation was observed for values of carbon pools in wetlands in the USA and Canada. Comparing the carbon pool function in European and African wetlands with the values observed in Mexican wetlands, both were similar for mangroves, marshes and flooded grasslands. Regarding the carbon pool reported in Africa, this was lesser than that detected in Mexican wetlands (Table 2).

Given the importance of carbon storage in Mexican wetlands, it is necessary to continue promoting the importance of their protection and conservation, their environmental services, and the economic value of these ecosystems. Some authors [29,30,65] in Mexico have established community participation works to rescue traditional uses of wetland resources and festivals on the importance of wetlands as awareness and appreciation strategies. In addition to the climate change threats to wetlands of North and Central America [66,67], it is time to pay attention to conserving the existing wetlands as natural treasures for the well-being of humans. 

## 4. Conclusions

Tropical wetlands are carbon-rich ecosystems. The Mexican carbon pool in the soil was reviewed according to the different wetland types, including swamps, mangroves, flooded grasslands, flooded palms and marshes. In Mexico, the mangrove has been the ecosystem with the most studies on carbon sequestration. This is probably due to the fact that they are the type of wetland that is protected under certain laws. New studies regarding different wetland ecosystems were found in which it was observed that swamps stored more carbon in the soil compared to other wetland types; however, the flooded grasslands and marshes presented a similar carbon pool to mangroves, so new public policies on protection and conservation of this type of wetland are needed. In the case of flooded palms, the average carbon pool of only three sites was 8 Kg C m^−2^; however, in addition to their importance and function as a carbon pool, such wetlands provide a social benefit due to the fruits of the palms in these ecosystems. Thus, this study claims that Mexican wetlands can be natural and cost-effective tools to store carbon in order to mitigate the effect of greenhouse gas emissions.

## Figures and Tables

**Table 1:** Carbon sequestration in Mexican wetland soils.

Forested Wetland Type	Site (Municipality or Area, State)	Carbon Stock (Kg C m^−2^)	Location in the Map (Figure 2)	Reference
Marshes	Tecolutla and Vega de Alatorre, Veracruz	25.9	D	Marín-Muñiz [31]
Marshes	Alto Lucero and Tecolutla, Veracruz	31.0	D	Campos [32]
Marshes	Veracruz, Tabasco/Campeche, Chiapas	110	D, G, E, F	Sjögersten et al. [33]
Marshes	Yucatán Peninsula	17.8	H	Adame et al. [34]
Marshes	Cuitzeo, Michoacán	16.8	K	Paredes-García et al. [35]
Marshes	La Encrucida, Biosphere Reserve, Chiapas	33.7	F	Adame et al. [36]
Marshes	Yucatán Peninsula	21.2	H	Morales-Ojeda et al. [37]
Marshes	Río Blanco, Veracruz	68	D	Hernández et al. [38]
Flooded grassland	Jamapa y Yagual, Veracruz	28	D	Hernández et al. [38]
Flooded grassland	Veracruz, Tabasco/Campeche, Chiapas	27.1	D, E, G, F	Sjögersten et al. [33]
Flooded grassland	Estero Dulce and Boquilla de Oro, Veracruz	30.6	D	Hernández et al. [25]
Mangrove	Yucatán Peninsula	28.0	H	Morales-Ojeda et al. [37]
Mangrove	Veracruz, Tabasco/Campeche, Chiapas	93	D	Sjögersten et al. [33]
Mangrove	Oaxaca and Guerrero	66.3	M, L	Herrera et al. [39]
Mangrove	Huimanguillo and Cárdenas, Tabasco	64.7	E	Moreno et al. [40]
Mangrove	Laguna de Términos, Campeche	25.2	G	Moreno-May et al. [41]
Mangrove	Carmen city, Campeche	11.7	G	Ceron-breton et al. [42]
Mangrove	Yucatán Peninsula	66.4	H	Adame et al. [34]
Mangrove	La Encrucida, Biosphere Reserve, Chiapas	78.5	F	Adame et al. [36]
Mangrove	Pantanos de Centla, Tabasco and Campeche	45.8	E, G	Kauffman et al. [43]
Mangrove	Vega de Alatorre, Veracruz	22	D	Hernández et al. [38]
Mangrove	La Encrucida, Biosphere Reserve, Chiapas	28.4	F	Adame and Fry. [44]
Mangroves	Alvarado, Veracruz	16	D	Moreno-Casasola et al. [45]
Mangrove	Tuxpan, Veracruz	14.7	D	Santiago [46]
Mangrove	Agua Brava Lagooon, Nayarit	4.2	C	Herrera-Silveira et al. [39]
Mangrove	Bahía Tóbari, Sonora	7.9	B	Bautista-Olivas et al. [47]
Mangrove	Cuyutlán, Colima	10.2	J	Herrera-Silveira et al. [39]
Mangrove	Nayarit	12	C	Valdés et al. [48]
Mangrove	La Paz Bay, Baja California	17.5	A	Ochoa-Gómez et al. [49]
Mangrove	Central coastal plain of Veracruz	37.5	D	Hernández and Junca-Gómez [50]
Mangrove	Paraíso Tabasco	20	E	Arias [51]
Mangrove	Península Yucatán	28.7	H	Gutiérrez-Mendoza and Herrera-Silveira[52]
Mangrove	Celestun, Yucatán	61.6	H	Herrera-Silveira et al. [53]
Mangrove	Nayarit	10	C	Valdés et al. [48]
Mangrove	Magdalena and Malandra bay. Baja California	22.5	A	Ezcurra et al. [54]
Mangrove	Sian Ka’an, Quintana Roo	45	I	Herrera-Silveira et al. [39]
Mangrove	Puerto Morelos, Yucatán	36	H	Herrera-Silveira et al. [39]
Mangrove	Aguiabampo, Sonora	3.5	B	Barreras-Apodaca et al. [55]
Mangrove	El Rabón, Nayarit	30	C	Castillo-Cruz and Rosa-Meza [56]
Mangrove	La Encrucijada, Chiapas	17.9	F	Barreras-Apodaca et al. [55]
Mangrove	Isla Arena, Campeche	30.5	G	Pech-Poot et al. [57]
Mangrove	Celestún, Yucatán	22.4	H	Pech-Poot et al. [57]
Mangrove	Cancún, Quintana Roo	26.4	I	Pech-Poot et al. [57]
Mangrove	La Encrucijada, Chiapas	6.3	F	Velázquez-Pérez et al.[58]
Mangrove	La Encrucijada, Chiapas	140		Sjögersten et al. [33]
Swamp	La Encrucida, Biosphere Reserve, Chiapas	72.2	F	Adame et al. [36]
Swamp	Jamapa, Veracruz	39	D	Hernández et al. [44]
Swamp	Alvarado, Veracruz	60	D	Moreno-Casasola et al. [45]
Swamp	Campeche y Tabasco	300	E, G	Sjögersten et al. [33]
Swamp	Tecolutla, Actopan, and Alto Lucero, Veracruz	45	D	Marín-Muñiz et al. [59]
Swamp	Alto Lucero and Tecolutla, Veracruz	52	D	Campos et al. [32]
Swamp	Tecolutla and Vega de Alatorre, Veracruz	35	D	Marín-Muñiz et al. [31]
Flooded Palm	Sian Ka’an, Quintana Roo	6.5	I	Alamilla, [60]
Flooded Palm	Alvarado, Veracruz	16	D	Moreno-Casasola et al. [45]
Flooded Palm	Jamapa, Veracruz	1.5	D	Sánchez [61]

**Table 2:** The carbon pool in the different wetland types of Mexico versus the carbon pool in other ecosystems and wetlands in other countries.

Ecosystem	Carbon Pool (Kg Cm^−^^2^)	Reference
Mexican terrestrial ecosystem	6.26	Vega-López [68].
Everette USA	7.81	Crooks et al. [69].
Clayoquot Sound marsh soils Canada	8.06	Chastain and Kohfeld[70].
African Salt Marshes	10.9	Raw et al. [71].
Okavango Delta, riverine marsh, Botswana, África	1.5	Bernal and Mitsch[24].
Wetlands of Europe	15–30	Abdul et al. [72].
Swamps	86.17	This study
Flooded grassland	28.57	
Mangroves	34.1	
Flooded palms	8.0	
Marshes	40.55	
